# Systematic genomic analysis of SARS-CoV-2 co-infections throughout the pandemic and segregation of the strains involved

**DOI:** 10.1186/s13073-023-01198-z

**Published:** 2023-07-24

**Authors:** Daniel Peñas-Utrilla, Laura Pérez-Lago, Andrea Molero-Salinas, Agustín Estévez, Amadeo Sanz, Marta Herranz, Carolina Martínez-Laperche, Cristina Andrés-Zayas, Cristina Veintimilla, Pilar Catalán, Roberto Alonso, Patricia Muñoz, Darío García de Viedma, on behalf of the Gregorio Marañón Microbiology-ID COVID 19 Study Group, Luis Alcalá, Teresa Aldámiz, Ana Álvarez-Uría, Elena Bermúdez, Emilio Bouza, Sergio Buenestado-Serrano, Almudena Burillo, Raquel Carrillo, Emilia Cercenado, Alejandro Cobos, Cristina Díez, Pilar Escribano, Chiara Fanciulli, Alicia Galar, Mª Dolores García, Paloma Gijón, Helmuth Guillén, Jesús Guinea, Álvaro Irigoyen, Martha Kestler, Juan Carlos López, Marina Machado, Mercedes Marín, Pablo Martín-Rabadán, Pedro Montilla, Belén Padilla, Rosalía Palomino-Cabrera, María Palomo, María Jesús Pérez-Granda, Leire Pérez, Elena Reigadas, Cristina Rincón, Belén Rodríguez, Sara Rodríguez, Cristina Rodríguez-Grande, Adriana Rojas, María Jesús Ruiz-Serrano, Carlos Sánchez, Mar Sánchez, Julia Serrano, Francisco Tejerina, Maricela Valerio, Lara Vesperinas, Teresa Vicente, Sofía de la Villa

**Affiliations:** 1 Servicio de Microbiología Clínica y Enfermedades Infecciosas, Hospital General Universitario Gregorio Marañón, C/Dr. Esquerdo 46, Madrid, 28007 Spain; 2 Instituto de Investigación Sanitaria Gregorio Marañón (IiSGM), Madrid, Spain; 3 Escuela de Doctorado, Universidad de Alcalá, Plaza de San Diego, S/N, Alcalá de Henares, Madrid, 28801 Spain; 4 Servicio de Oncohematología, Gregorio Marañón General University Hospital, Madrid, Spain; 5 Genomics Unit, Gregorio Marañón General University Hospital, Madrid, Spain; 6 CIBER de Enfermedades Respiratorias, Instituto de Salud Carlos III, Madrid, Spain; 7 Departamento de Medicina, Universidad Complutense, Madrid, Spain; 8 Centro de Investigación Biomédica en Red (CIBER) de Enfermedades Infecciosas (CIBERINFEC), Madrid, Spain

**Keywords:** SARS-CoV-2, Co-infections, WGS, Segregation

## Abstract

**Background:**

SARS-CoV-2 recombinants involving the divergent Delta and Omicron lineages have been described, and one of them, “Kraken” (XBB.1.5), has recently been a matter of concern. Recombination requires the coexistence of two SARS-CoV-2 strains in the same individual. Only a limited number of studies have focused on the identification of co-infections and are restricted to co-infections involving the Delta/Omicron lineages.

**Methods:**

We performed a systematic identification of SARS-CoV-2 co-infections throughout the pandemic (7609 different patients sequenced), not biassed towards the involvement of highly divergent lineages. Through a comprehensive set of validations based on the distribution of allelic frequencies, phylogenetic consistency, re-sequencing, host genetic analysis and contextual epidemiological analysis, these co-infections were robustly assigned.

**Results:**

Fourteen (0.18%) co-infections with ≥ 8 heterozygous calls (8–85 HZs) were identified. Co-infections were identified throughout the pandemic and involved an equal proportion of strains from different lineages/sublineages (including pre-Alpha variants, Delta and Omicron) or strains from the same lineage. Co-infected cases were mainly unvaccinated, with mild or asymptomatic clinical presentation, and most were at risk of overexposure associated with the healthcare environment. Strain segregation enabled integration of sequences to clarify nosocomial outbreaks where analysis had been impaired due to co-infection.

**Conclusions:**

Co-infection cases were identified throughout the pandemic, not just in the time periods when highly divergent lineages were co-circulating. Co-infections involving different lineages or strains from the same lineage were occurring in the same proportion. Most cases were mild, did not require medical assistance and were not vaccinated, and a large proportion were associated with the hospital environment.

**Supplementary Information:**

The online version contains supplementary material available at 10.1186/s13073-023-01198-z.

## Background

Population-level genomic analysis has been essential to track SARS-CoV-2 transmission with greater precision and determine the evolutionary dynamics leading to the emergence of progressively more successful variants [1–3]. Within-patient whole-genome sequencing has also provided a better understanding of infection, leading to very precise identification of re-infections [4, 5] and characterization of diversity in long-term persistent cases [6, 7].

However, one aspect has not yet been adequately addressed, namely, an analysis of co-infections with more than one SARS-CoV-2 strain. The recent description of recombinants between the emerging, divergent Delta and Omicron BA.1 and BA.2 variants [8–10] has raised interest in the identification of co-infections, a prerequisite for the occurrence of recombinant events.

Most studies focused on co-infections are limited to analyses involving the recent divergent lineages and are therefore restricted to the latter stages of the pandemic when they were co-circulating [11–13]. Very few studies offer data on co-infections in the COVID-19 waves that preceded the emergence of the Delta/Omicron lineages [14, 15].

Our study is a systematic analysis of SARS-CoV-2 co-infections in an entire population, all the COVID-19 cases diagnosed in the population covered by our hospital, over the entire course of the pandemic. Our identification scheme is not based on the selection of a small set of lineage-marker SNPs to identify co-infections targeting a limited number of lineages, but on the unbiased identification of co-infecting strains of any lineage, even the same one. A segregation pipeline was also developed to reconstruct individual sequences from the two strains involved in each co-infection for further virological/epidemiological analysis.

## Methods

### Patients and materials

The study included all the 7609 different COVID-19 patients diagnosed in our institution between March 2020 and September 2022 and with good quality sequences (≥ 90% of the genome covered > 30 ×). The material for analysis corresponded to remnants of nasopharyngeal swabs taken for diagnostic purposes. RNA was extracted and purified from 300 μL of nasopharyngeal exudate in a KingFisher (Thermo Fisher Scientific) instrument. The criterion for selection of positive specimens for study was a RT-PCR (TaqPath COVID-19 CE-IVD RT-PCR kit; Thermo Fisher Scientific) cycle threshold (*C*_*t*_) value < 32 for the nucleocapsid gene.

### Illumina sequencing

Sixteen microlitres of RNA was used as a template for reverse transcription using the LunaScript RT SuperMix Kit (New England BioLabs). Whole-genome amplification of the coronavirus was performed with the Artic nCoV-2019 V3, V4 and V4.1 panel of primers, sequentially, as soon as they were launched (Integrated DNA Technologies, artic.network/ncov-2019) and Q5 Hot Start DNA polymerase (New England BioLabs). Libraries were prepared using the DNA Prep Kit (Illumina), following the manufacturer’s instructions, and were quantified with the Quantus Fluorometer (Promega), before being pooled at equimolar concentrations (4 nM). They were then sequenced in 96 pooled libraries on a MiSeq V2 flow cell (300 cycle format).

### Artificial in vitro mixtures

To validate the efficiency of our pipeline to correctly identify co-infections, to call HZ positions and to finally segregate the co-infecting strains, we prepared artificially simulated co-infections by performing in vitro mixtures. We selected one representative of the following lineages: Alpha, Delta and Omicron (BA.2 and BA.5). We prepared three mixes of two strains differing in a high, intermediate or low number of SNPs between them. Each pair of strains were mixed in different ratios: 1:0 (100–0%), 1:1 (50–50%), 2:1 (66–33%), 3:1 (75–25%), 9:1 (90–10%) and 0:1 (0–100%). To better control the relative proportions between the strains in the artificial mixtures, we combined the respective amplicon pools immediately after having been quantified (Quantus Fluorometer; Promega), and then, we proceeded with the library preparation.

### Bioinformatic analysis

An in-house bioinformatics pipeline was applied to analyse sequencing reads (https://github.com/MG-IiSGM/SARS_COVinfections). Adapters and low-quality regions were processed from paired reads using fastp (version 0.20.1 [16]). Quality control was assessed with fastQC (version v0.11.9, https://github.com/s-andrews/FastQC). Good-quality reads were mapped with BWA (version 0.7.17-r1188 [17]) to the Wuhan-1 SARS-CoV-2 reference sequence (GenBank accession no. NC_045512.2); IVar (version 1.3.1 [18]) was used to call variants, and pangolin (version 4.1.2 [19]) was used for lineage annotation.

### Detection of co-infection candidates

For co-infection detection, only SNPs called with IVar in samples with a good coverage (≥ 90% of the genome covered at a depth of > 30 ×) were considered. The co-infection detection pipeline uses a Python script that focuses on SNP call frequency. In each specimen, the pipeline identifies genome positions where alternate allele frequency is ≥ 85% (homozygous SNPs), positions where two alleles co-exists and the higher alternative allele frequency is between 15 and 85% (heterozygous SNPs) and positions where the alternative allele is in a proportion of ≤ 15% and is considered to be background sequencing noise and therefore ruled out. For heterozygous positions, the pipeline computes the following: (1) the mean proportion of major alleles (MHP), (2) the standard deviation of the MHP (SHP) and (3) the percentage of SNPs with a heterozygous proportion of the major allele found within the MHP ± (SHP + 1.5%) (SWS). To minimize the selection of cases with HZ content due to sequencing background noise, those with MHP < 75% were selected as candidates for co-infection. Therefore, we established a minimum average proportion of 25% for the minor strain in a co-infection, while allowing for heterozygous (HZ) calls to reach a minimum proportion of 15%.

Since the relative allele frequencies in a co-infection should be consistent across all HZ calls, we considered as co-candidates for co-infection those with (1) mean SHP ≤ 8% and (2) ≥ 70% of the HZ calls within that std (SWS). Both metric values derived from the behaviour of HZ calls observed in silico when testing controlled mixes sequences from different lineages (Alpha, Delta and Omicron) at various proportions (50–50%, 65–35%, 75–25) (the fastq files generated for these in silico mixes are available upon request).

### Segregation of co-infecting strains

Segregation of the two strains involved in a co-infection was confidently performed when the mean HZ frequency was > 60%. Two fasta sequences were generated for the minority (1) and majority (2) strains, respectively. For specimens with several SNPs with a HZ frequency between 45 and 55%, segregation may give rise to erroneous sequences. For these positions, a consensus base was generated corresponding to the capture of both alleles. Lineage annotation with pangolin was performed on both segregated strains.

### Phylogenetic consistency of segregated strains

For the analysis of phylogenetic consistency, multiple sequence alignment was performed with the MAFFT aligner (https://mafft.cbrc.jp/alignment/software/). The snipit program (https://github.com/aineniamh/snipit) was used for MSA visualization, and a phylogenetic tree was constructed with iqtree (http://www.iqtree.org/) and visualized with Microreact (https://microreact.org/).

### Host genetic analysis

Short tandem repeat analysis (STR)-PCR (Mentype^®^ Chimera^®^ Biotype, Germany) was applied for human identity testing, for which the same specimens used for SARS-CoV-2 genome sequencing were employed. We examined 12 non-coding STR *loci* and the gender-specific amelogenin *locus*, labelled with three different dyes (6-FAM™, BTG or BTY). PCR was performed with 0.2–1 ng of genomic DNA with the Mentype^®^ Chimera^®^ PCR amplification kit (Biotype, Germany), the GeneAmp^®^ PCR System 9700 Thermal Cycler (Applied Biosystems) and subsequent analysis by capillary electrophoresis in a 3130*xl* Genetic Analyzer (Applied Biosystems), as recommended by the manufacturer.

## Results

### Determining the magnitude of SNP-based diversity throughout the pandemic

Since SARS-CoV-2 co-infections with two strains give rise to sequences with heterozygous (HZ) calls, we focused on determining the threshold number of HZ positions for suspecting the presence of two strains simultaneously. To do this, using pairwise alignment of sequences, we first defined the minimum diversity observed between any two sequences during the pandemic (March 2020 to September 2022) divided into 2-month time frames.

Only consolidated SNPs of high quality (COV > 30 ×) were considered. As expected, as the pandemic progressed, there was a tendency for the number of SNPs acquired to increase over time (Table 1). However, there were periods of marked reductions in diversity, which corresponded to the introduction of a new lineage into the population that outcompeted the previous ones before its within-lineage diversity increased. Pairwise diversity between any two sequences fluctuated depending on whether or not different lineages were co-circulating during the same 2-month period. In summary, diversity ranged from 8 SNPs at the beginning of the pandemic (February to March 2020) to 50 when B.1.1.7 and AY.43 co-circulated (June to July 2021). The mean within-lineage diversity was 9 SNPs ± 3.78, with the lowest and highest values being obtained for BA.1.17 (4.9 ± 2.14) and B.1.1.7 (13.11 ± 3.56). Taking all these data together, we determined 8 SNPs as the threshold of diversity—and hence the number of HZ calls—for suspecting the presence of two SARS-CoV-2 strains infecting the same individual.

**Table 1:** The mean total number of acquired SNPs and mean pairwise distances in the sequences obtained from our population in each 2-month period of the pandemic

Date	Mean number of acquired SNPs	Mean pairwise distance
2020 February/2020 March	5.86 ± 2.33	8.23 ± 4.63
2020 April/2020 May	7.34 ± 2.41	9.6 ± 4.91
2020 June/2020 July	11.74 ± 4.38	17.79 ± 6.64
2020 August/2020 September	14.28 ± 2.15	8.73 ± 5.12
2020 October/2020 November	17.17 ± 3.91	12.88 ± 6.54
2020 December/2021 January	22.44 ± 6.18	29.53 ± 14.26
2021 February/2021 March	29.73 ± 6.65	28.89 ± 16.17
2021 April/2021 May	34.62 ± 5.12	37.17 ± 18.31
2021 June/2021 July	36.05 ± 5.52	50.89 ± 21.31
2021 August/2021 September	37.93 ± 6.15	22.15 ± 12.42
2021 October/2021 November	42.21 ± 7.21	23.36 ± 9.35
2021 December/2022 January	53.68 ± 6.16	31.22 ± 30.02
2022 February/2022 March	63.19 ± 7.46	33.11 ± 23.35
2022 April/2022 May	70.63 ± 5.87	12.65 ± 5.33
2022 June/2022 July	70.72 ± 4.63	15.49 ± 6.99
2022 August/2022 September	73.38 ± 4.15	16.81 ± 6.02

### Experimental in vitro validation of the co-infection pipeline

We conducted an in vitro validation experiment to assess the performance of our co-infection pipeline using controlled artificial mixtures. We combined in vitro specimens from various lineages and mixed them at different proportions. The mixes comprised of Delta and Alpha, Omicron BA.5 and Omicron BA.2, and Omicron BA.5 and Omicron BA.5 lineages to lead to mixes differing in a high (64 SNPS), intermediate (16 SNPs) or low (9 SNPs) number of SNPs, respectively (Additional file 1: Tables S1, S2, S3). The strategy employed for generating the mixes involved mixing the amplicon pools after DNA quantification to ensure the theoretical proportion of each specimen. Sequencing statistics demonstrated that the generated mixes were of high quality (≥ 90% of the genome covered > 30 ×).

When we applied the co-infection pipeline to the mixtures, a close agreement between the expected majority proportion and number of HZ calls between the experimental values and the expected data was obtained (Additional file 1: Tables S1, S2, S3). In the negative controls, no HZ calls were observed for each of the 1:0 and 0:1 mixes. For the 9:1 mix, either no HZ calls (for Omicron mixes) or a small number of HZ calls (< 8 and therefore insufficient to be considered as co-infection) were observed (Additional file 1: Tables S1, S2, S3). This observation supported that we could not identify co-infections when the minority strain was so underrepresented. As our threshold to consider HZs to suspect co-infection is set at 85%, in this, 9:1 control co-infection could not be considered. For the positive controls 1:1, 2:1 or 3:1, our pipeline successfully identified co-infections, and all them met our criteria to assure a consistent homogeneous distribution of HZ allele frequencies. In all cases, the standard deviation (SD) of HZ major alleles was less than 8%, and the proportion of HZ calls within this narrow SD was in all cases > 70%.

In terms of segregation, our pipeline, as expected, failed to confidently assign lineages for the segregated sequences in the 1:1 control, as HZ calls were close to the 50:50 threshold, which is responsible for failures in allele assignments and consequently leads to low-confident segregation. However, for the 2:1 and 3:1 positive controls, lineage assignment was consistent with the expected results, and the conflict in the assignation was inexistent. Additionally, our results indicate that no significant amplification bias was observed for any lineage during the generation of mixtures. Therefore, the proportion of each strain involved in a co-infection as determined by our pipeline would potentially represent the actual proportion of both strains.

### Identification of candidates for SARS-CoV-2 co-infection and subsequent validation

**Fig. 1:**
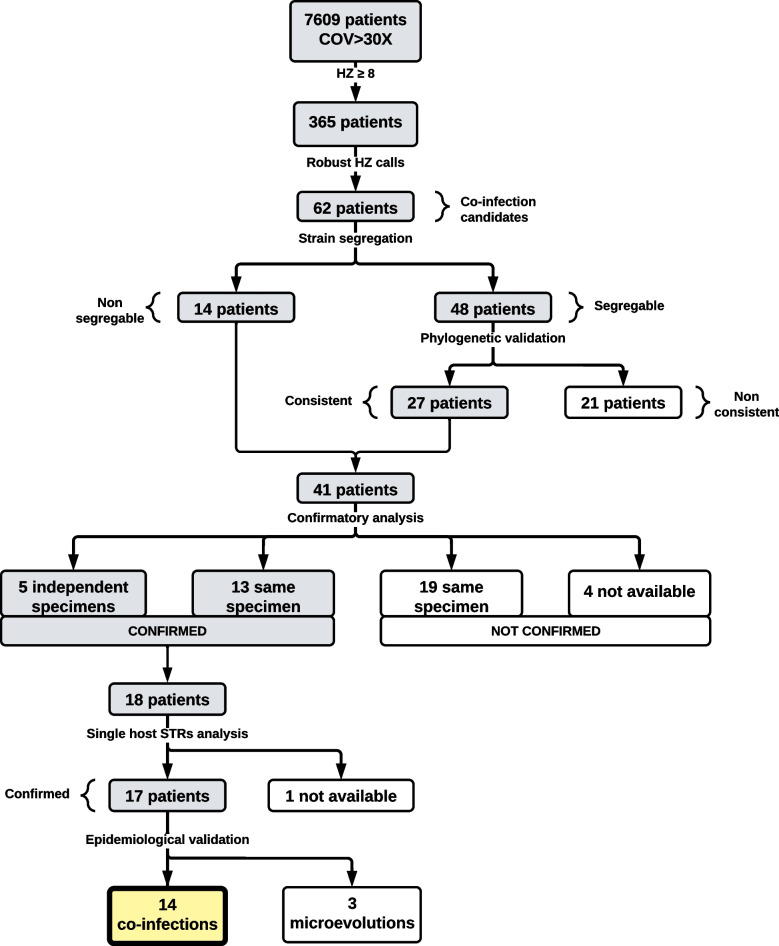
Flowchart. Identification of candidates for co-infection and validation procedures. HZ, heterozygous; STR, short tandem repeats

Among the sequences obtained from 7609 different COVID-19 patients between March 2020 and September 2022 (≥ 90% of the genome covered > 30 ×), 365 (4.8%) showed ≥ 8 HZ calls (Fig. 1). To increase confidence that these corresponded to co-infected patients, three requirements were applied (the “Methods” section). First, to rule out the possibility that the HZ call was due to sequencing noise, the mean frequency of the majority allele in HZ calls had to be < 0.75 and standard deviation ≤ 0.08. Second, to maximize the expected consistency for HZ calls due to co-infecting strains, we required that the frequencies of ≥ 70% of HZ calls fall within the interval defined by the mean frequency of the HZ calls ± 0.095; 62 of the sequences satisfied this requirement (Fig. 1).

The next step was to validate whether or not the 62 candidates corresponded to co-infections. For this validation, we applied a series of filters, based either on (i) population phylogenetic analysis, (ii) intra-patient analysis and (iii) epidemiological analysis.

### Phylogenetic validation

To assess the phylogenetic consistency of the candidates for co-infection, the two co-infecting strains in each case were first segregated using bioinformatics. Once segregated, we assessed whether they were consistently positioned on the phylogenetic trees containing the sequences of strains circulating in our population 4 weeks before and after the diagnosis of each putative co-infection (average number for each analysis 256 sequences).

To segregate the sequences, we designed a pipeline to determine the sequences corresponding to the majority and minority co-infecting strains harbouring the majority and minority alleles in the HZ calls, respectively. The pipeline was able to segregate co-occurring sequences in 48 cases using the relative proportions of alternative alleles in the HZ calls (one allele with a frequency of > 0.6) to identify the majority and minority strains (the “Methods” section). In 14 cases, the relative frequencies were so close to 50:50 that it was not possible to segregate the coexisting sequences (Fig. 1).

**Fig. 2:**
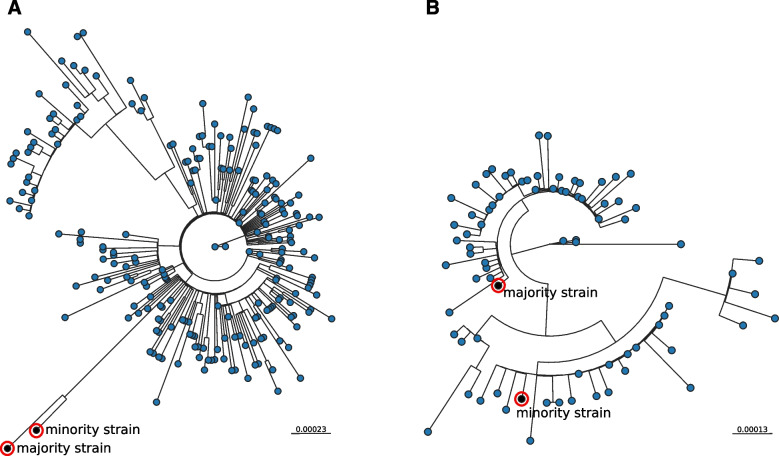
Phylogenetic validation of co-infection candidates. Phylogenetic tree including the sequences from segregated co-infecting strains and sequences obtained from all SARS-CoV-2 cases diagnosed 4 weeks before and after diagnosis of the candidate for co-infection (scale bar: substitutions per site). We present two representative examples of **a** an epidemiologically inconsistent case (patient 55 in Additional file 1: Table S4). The two segregated sequences from the co-infected candidate were located closer to each other, including sharing the same branch, than to any other circulating strain, and **b** an epidemiologically consistent case (patient 2 in Additional file 1: Table S4). The two segregated sequences are interspersed on the tree. Majority- and minority-segregated strains are represented by a black dot in a red circle

After incorporating the segregated sequences of the 48 co-infection candidates into the same phylogenetic tree together with all the circulating strain sequences in the same 2-month period as the case diagnosis, the candidates could be divided into two groups, based on the phylogenetic distribution of the segregated strains: (i) 21 cases showed inconsistent behaviour, with the two segregated sequences located closer to each other (even sharing a single branch) than to any other circulating strain (Fig. 2a), and (ii) 27 cases showed a consistent pattern, with the two segregated sequences being interspersed throughout the tree (Fig. 2b). Most of the candidates with inconsistent phylogenetic behaviour, which were eliminated from the study, corresponded to specimens with higher *C*_*t*_ values (Additional file 1: Table S4). This inconsistent behaviour could be due to poor sample quality or to some degree of background sequencing noise.

### Intra-patient confirmation of co-infections and ruling out cross-contamination

We then applied a second validation criterion to 27 candidates that showed consistent phylogenetic behaviour after strain segregation, together with the 14 candidates with co-infecting strains that could not be segregated because the proportion was so close to 50:50 (Fig. 1). In these 41 cases, validation of co-infection was sought by confirming the presence of the same co-infecting strains, either by re-sequencing the same specimen or by detecting the same co-infection in a separate sample from the same patient. In four candidates, no additional material was available for this validation and they were discarded from the study. In another five cases, separate samples were available and the same two co-infecting strains were detected in all five. In the remaining 32 candidates, the same specimens were re-extracted and re-sequenced, and in 13 of them, the same two co-infecting strains were detected once again. In most of them, a good correlation between the relative frequencies of the two specimens (Pearson correlation coefficient = 0.89, *p*-value = 0.0002) was found. However, only one of the two strains was identified in the 19 remaining candidates for co-infection, and these were considered to be due to inter-specimen cross-contamination when managing the specimens or at any step along library preparations for sequencing them. The likely source of contamination was traced in 12 of the 19 cases; once the co-occurring strains had been segregated, we were able to identify another specimen with the same sequence sharing the same sequencing run (0 SNPs).

Finally, to further validate the 18 co-infection candidates in which the same co-infecting strains were redetected, human identity testing was performed on the human DNA content present in the same specimen that had been used for SARS-CoV-2 sequencing (Fig. 1). In one case, no remaining material was available, but short tandem repeat analysis of the other 17 samples indicated that the human material in every specimen corresponded to a single individual. This allowed us to rule out that the presence of two sequences was due to the mishandling of specimens resulting in cross-contamination prior to sequencing.

### Epidemiological validation

As our candidates for co-infection were distributed throughout the entire COVID-19 pandemic period (Additional file 1: Table S4), we included the epidemiological scenario at the time of diagnosis of each case as a new validation stage. The ability to segregate co-infecting strains when the relative frequency of one of them was > 0.6 enabled us to assign lineage in 10 of the 17 candidates. In another three cases, after re-sequencing or obtaining another specimen from the same patient, the relative proportion of one of the co-infecting strains increased and could therefore be used as a reference for segregation. Finally, in four cases, complete genome segregation was not feasible, and we obtained instead a consensus sequence that made it possible to assign lineage. In total, seven co-infections involved strains from different lineages or sublineages, and the remaining ten involved different strains from the same sublineage.

**Fig. 3:**
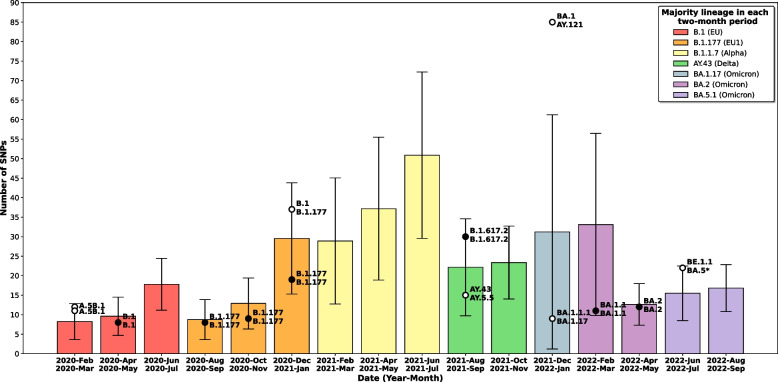
Epidemiological validation of the co-infections. Each bar corresponds to the mean pairwise SNP distance and standard deviation between any two circulating strains in our population in each 2-month period during the pandemic. Each black dot or open circle corresponds to a co-infected case and is plotted by taking into account the number of SNPs between co-infecting strains. Co-infections involving strains from the same or different lineage/sublineages are shown as black dots or open circles, respectively. The majority and minority lineages involved in each co-infection are shown top and bottom, respectively (except for the first 2-month period where they are shown as left and right). The colour of the bar indicates the majority lineage in each period (see legend)

Once the co-infecting strains had been segregated, we then undertook a more detailed analysis of the strains involved according to their consistency with the epidemiological scenario at the time of diagnosis of each case. We first evaluated whether the diversity (number of SNPs) between the segregated sequences in each co-infected case was consistent with the measured diversity for circulating strains (within a 2-month time window) in our population. In all but one case, diversity among co-infecting strains was within the range of diversity recorded in the population (Fig. 3). The only case with higher diversity (85 SNPs; case 11) corresponded to a Delta/Omicron co-infection and occurred at the time (December 2021) when one of the lineages was being replaced by the other. Secondly, in all cases, we evaluated whether the lineages involved in each co-infection matched those circulating at the corresponding point of the pandemic. In 14 cases, the lineages involved corresponded to the majority circulating variants. In the remaining three, the lineages involved were not circulating at the time of their diagnosis; B.1.1/B.1.1 (case 40), B.1.177/B.1.177 (case 41) and BA.2 /BA.2 (case 62) (Additional file 1: Table S4) co-infections at the beginning of October 2021, December 2021 and September 2022 were found, when AY.43, BA.1.17 and BA.5.1, respectively, were the main circulating lineages. The clinical charts of these cases were reviewed, and all three patients were severely immunosuppressed with long-term COVID-19. Two cases had a diagnosis of non-Hodgkin’s lymphoma with persistently positive SARS-CoV-2 RT-PCRs months before the tested specimen (case 40 since October 2020; case 41 since November 2020), and the remaining case was a heart transplant recipient with active immunosuppression and long-term COVID-19 for at least 3 months.

In these three cases, instead of co-infection, we considered the alternative hypothesis of microevolution of a long-term persistent case of infection leading to (i) sufficient acquisition of diversity to exceed our threshold (> 8 SNPs) for considering co-infection and (ii) within-patient maintenance of a lineage beyond the time of circulation. Through an in-depth analysis of the sequences from their specimens, we were able to identify a consensus core genome shared by all specimens (including lineage marker SNPs and several strain-specific SNPs) and the occurrence of additional SNPs not shared by all the specimens, consistent with marked acquisition of diversity. In one of the cases (case 40), we were able to perform a more exhaustive analysis, as we had 8 specimens available covering an 8-month period (from June 2021 to January 2022; Additional file 1: Fig. S1). These three cases were discarded therefore, and we finally deemed 14 cases to be robustly confirmed SARS-CoV-2 co-infections (Table 2), supported by the sequential series of validation filters. Seven co-infections involved different lineages/sublineages (corresponding to the pre-Alpha, Delta and Omicron variants), and the remaining seven involved strains from the same sublineage (pre-Alpha or Omicron).

**Table 2:** Patient sequence/specimen characteristics of validated SARS-CoV-2 co- infections. Bold rows correspond to the specimen that revealed the co-infection event. Non-bold rows correspond to replicates (-BIS) or independent specimens from the same patient. ^*^Lineage assigned after discarding segregated positions with lower confidence. %: Percentage, HZ: heterozygous, COV: Coverage, STD: Standard deviation

**Patient**	**Sample**	**Gen N2 Ct**	**% COV>30X**	**HZ calls (N)**	**mean HZ proportion**	**std HZ proportion**	**% SNPs within STD**	**Minority lineage**	**Majority lineage**	**Sample Date**
1	** ERS14369070 **	** n.a **	**99.69**	**12**	**0.66**	**0.04**	**83**	**A.5**	**B.1**	**2020-03-09**
	ERS14369071 (-BIS)	n.a	99.58	12	0.67	0.02	83	A.5	B.1	2020-03-09
2	** ERS14369072 **	** n.a **	**99.08**	**11**	**0.64**	**0.03**	**91**	**A.5**	**B.1**	**2020-03-09**
	ERS14369073 (-BIS)	n.a	99.58	12	0.63	0.07	92	A.5	B.1	2020-03-09
3	** ERS14369074 **	** n.a **	**99.02**	**8**	**0.73**	**0.03**	**88**	**B.1**	**B.1**	**2020-04-08**
	ERS14369075 (-BIS)	n.a	99.1	8	0.66	0.05	88	B.1	B.1	2020-04-08
4	** ERS14369076 **	**20**	**99.66**	**8**	**0.66**	**0.04**	**75**	**B.1.177**	**B.1.177**	**2020-08-11**
	ERS14369077 (-BIS)	20	99.58	8	0.67	0.03	88	B.1.177	B.1.177	2020-08-11
5	** ERS14369078 **	**25**	**98.71**	**9**	**0.54**	**0.03**	**100**	**B.1.177**	**B.1.177**	**2020-11-01**
	ERS14369079 (-BIS)	25	99.02	9	0.55	0.04	89	B.1.177	B.1.177	2020-11-01
6	** ERS14369080 **	**31**	**99.02**	**37**	**0.6**	**0.05**	**76**	**B.1.177**	**B.1**	**2021-01-18**
	ERS14369081	14	98.97	37	0.76	0.06	89	B.1	B.1.177	2021-01-27
	ERS14369082	22	99.58	1	0.52	0	100	B.1.177	B.1.177	2021-02-01
7	ERS14369083	16	99.58	19	0.78	0.03	95	B.1.177	B.1.177	2021-01-21
	** ERS14369084 **	**18**	**99.67**	**19**	**0.61**	**0.04**	**84**	**B.1.177**	**B.1.177**	**2021-01-21**
8	** ERS14369085 **	**25**	**98.79**	**30**	**0.55**	**0.06**	**90**	**B.1.617.2**	**B.1.617.2**	**2021-08-08**
	ERS14369086 (-BIS)	25	99.56	32	0.57	0.04	91	B.1.617.2	B.1.617.2	2021-08-08
9	** ERS14369087 **	**26**	**99.67**	**15**	**0.58**	**0.03**	**93**	**AY.5.5**	**AY.43**	**2021-08-25**
	ERS14369088 (-BIS)	26	99.66	15	0.64	0.03	73	AY.5.5	AY.43	2021-08-25
10	** ERS14369089 **	**12**	**98.44**	**9**	**0.71**	**0.03**	**89**	**BA.1.17**	**BA.1.1.1**	**2021-12-14**
	ERS14369090 (-BIS)	12	98.41	9	0.74	0.05	89	BA.1.17	BA.1.1.1	2021-12-14
11	** ERS14369091 **	**16**	**99.62**	**85**	**0.71**	**0.07**	**74**	**AY.121**	**BA.1**	**2021-12-17**
	ERS14369092 (-BIS)	16	99.58	89	0.76	0.04	79	AY.121	BA.1	2021-12-17
	ERS14369093	29	99.58	1	0.81	0	100	AY.121	AY.121	2021-12-23
12	ERS14369094	18	98.73	9	0.7	0.07	67	BA.1.1	BA.1.1	2022-02-09
	** ERS14369095 **	**31**	**98.91**	**11**	**0.73**	**0.04**	**73**	**BA.1.1**	**BA.1.1**	**2022-02-11**
13	** ERS14369096 **	**14**	**99.3**	**12**	**0.57**	**0.07**	**92**	**BA.2**	**BA.2**	**2022-05-12**
	ERS14369097	23	98.69	11	0.53	0.02	91	BA.2	BA.2	2022-05-17
14	** ERS14369098 **	**18**	**98.61**	**22**	**0.7**	**0.05**	**82**	BA.5*	**BE.1.1**	**2022-06-27**
	ERS14369099	28	95	14	0.79	0.05	86	BA.5	BE.1.1	2022-07-08

### Analysis of intra-patient co-infection dynamics

**Fig. 4:**
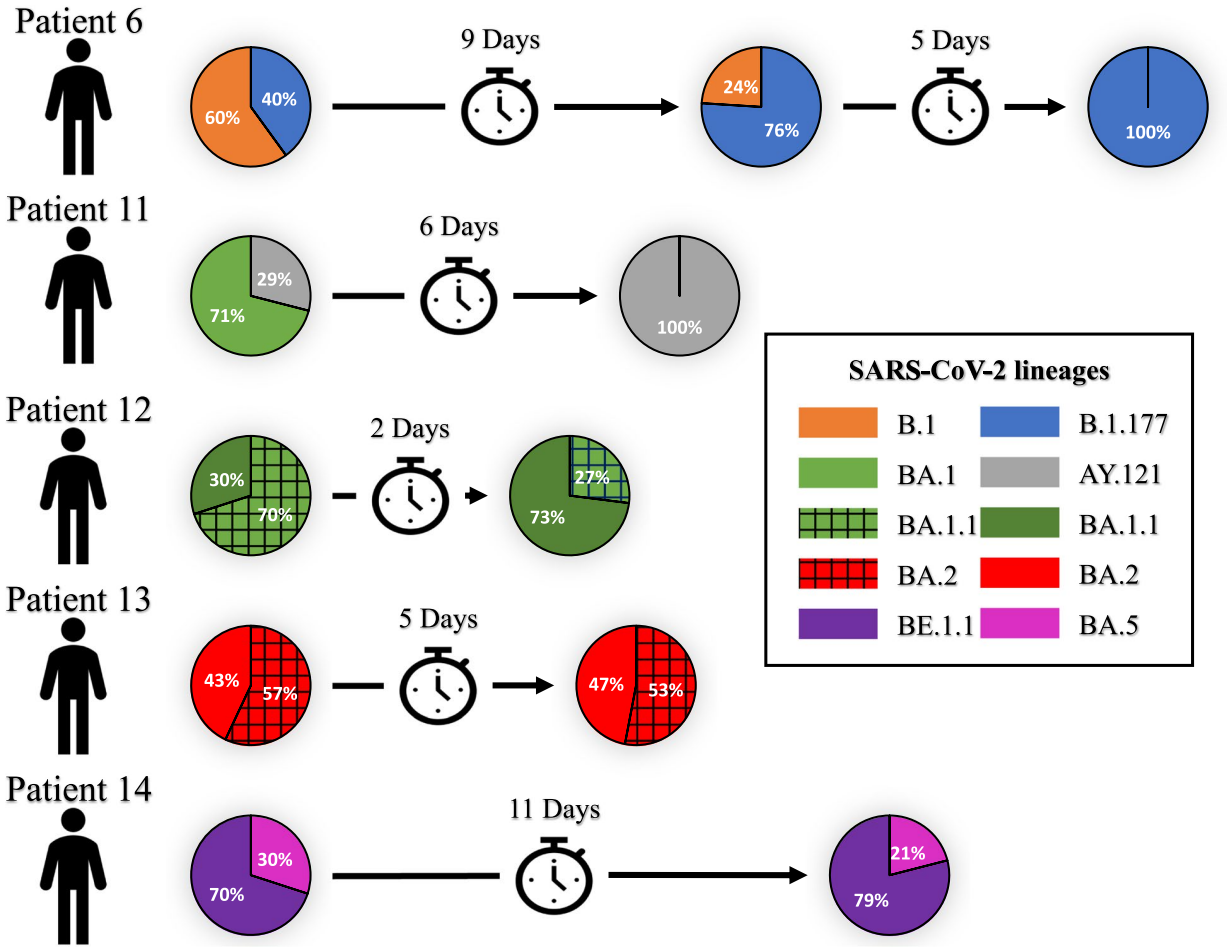
Chronological within-host dynamics of co-infecting strains for the five patients with more than one specimen available from different periods. Pie charts depict the relative proportion of each co-infecting strain at each sampling time point. The colour of each pie sector corresponds to the lineages involved in each co-infection (see legend)

In 5 of the co-infected patients (involving 8 different lineages: B.1, B.1.177, AY.121, BA.1, BA.1.1, BA.2, BA.5, BE.1.1), one or more additional positive specimens from a different sampling day were available. This enabled us to characterize the chronological dynamics of the co-infecting strains. In four cases, co-infection was also detected in the second specimen (2–14 days apart, Fig. 4). In two of these (patients 13 and 14, Fig. 4), the relative proportions of the co-infecting strains observed in the first specimen tested (0.57 and 0.7) were maintained in the second confirmatory specimen (0.53 and 0.79, respectively). In the other two cases (patients 6 and 12, Fig. 4), we noticed that the relative proportions of the co-infecting strains changed markedly, with the minority strain (B.1.177 and BA.1.1 lineages) becoming the majority one, and vice versa. In patients 6 and 11 (Fig. 4), one of the co-infecting strains (B.1 and BA.1) was outcompeted (by B.1.177 and AY.121, respectively) 5 and 6 days later; in both of them, the strain that eventually emerged as dominant was initially a minority strain (Fig. 4).

### Clinical analysis

The median age of the 14 patients with co-infection was 51 years, eight were males (57.1%) and six were females (42.9%; Table 3). Half of them (7/14) had no medical history of interest. Heart disease (4/14) and diabetes (4/14) were the most prevalent conditions. In terms of severity of COVID-19 episodes, eight (57.1%) were mild, four (28.6%) were severe and one (7.1%) was asymptomatic. In terms of the need for healthcare, two (14.3%) were treated exclusively in the emergency department, three (21.4%) required hospital admission for COVID-19 and one (7.1%) patient required admission to the intensive care unit. In terms of vaccination, nine (64.3%) were unvaccinated, although in seven of these cases, the reason for this was that vaccination was not yet available at the time of infection (episodes prior to February 2021). Three patients died due to SARS-CoV-2 infection: all were over 75 years of age, had multiple comorbidities and were not vaccinated. Eight patients (57.1%) were healthcare-related, three either to nosocomial outbreaks or to nosocomial acquisition and four others were healthcare workers at our hospital.

**Table 3:** Clinical characteristics of patients with SARS-CoV-2 co-infections

*N*: 14	
**Demographics**	
**Age** (median/IQR)	51/45–76
**Gender**	*Total/%*
Male	8/57.1
Female	6/42.9
**Severity**^**a/b**^	
Asymptomatic	1/7.1
Mild	8/57.1
Moderate	0/0.0
Severe	4/28.6
**Need for healthcare**	
Emergency	2/14.3
Hospital admission	6/42.9
Hospital admission for COVID-19	3/21.4
ICU	1/7.1
ICU for COVID-19	1/7.1
**Previous diseases**	
None of interest	7/50.0
Heart disease	4/28.6
Oncological	3/21.4
Diabetes	4/28.6
High blood pressure	3/21.4
Overweight/obesity	3/21.4
COPD	1/7.1
Ictus	1/7.1
Autoimmune	1/7.1
Chronic kidney disease	1/7.1
HIV infection	0/0.0
**Antiviral treatment**^**c**^	
Lopinavir/ritonavir	3/21.4
Hydroxychloroquine	2/14.3
Interferon	2/14.3
Dexamethasone	1/7.1
**Exitus by COVID-19**	3/21.4
**Vaccination and serology status**^**d**^	
Complete vaccination schedule	5/35.7
Incomplete vaccination schedule	0/0.0
Unvaccinated	9/64.3
Previously positive serology for SARS-CoV-2	2/14.3
Previously negative serology for SARS-CoV-2	0/0.0
Serology not available	12/85.7
**Risk of overexposure**	
Healthcare professionals	4/28.6
Involvement in nosocomial outbreak	4/28.6

^a^We have no clinical information on the COVID-19 episode in one patient

^b^The definition of patient severity has been organized according to the following criteria: Mild—general malaise, cough, diarrhoea, headache, fever, anosmia, myalgias, rhinorrhoea; moderate—previous symptoms plus dyspnoea, mild respiratory failure, or unilateral pneumonia; severe—previous symptoms plus bilateral pneumonia or severe respiratory failure

^c^Patients with COVID-19 episode who received lopinavir/ritonavir, hydroxychloroquine and interferon were from March and April 2020, when it was considered the standard of care in our centre

^d^The high proportion of unvaccinated patients is largely due to episodes of COVID-19 reported in 2020 and/or early 2021 (7/9), when the vaccine was not yet available

### Epidemiological exploitation of segregated strains

**Fig. 5:**
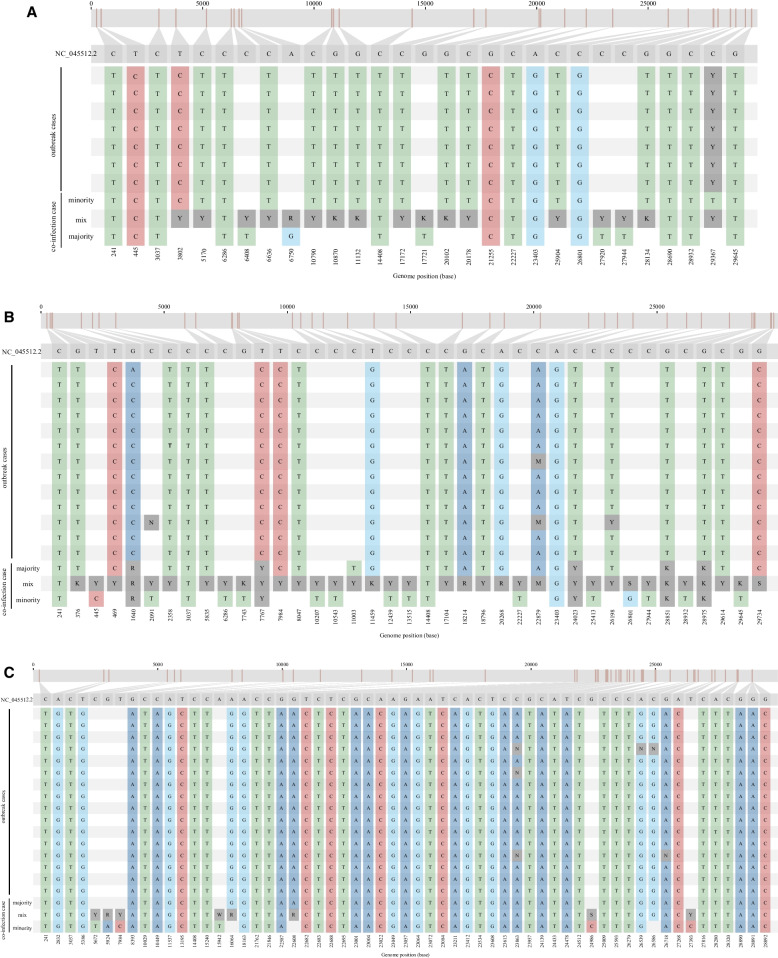
Multiple alignment of sequences from cases involved in three nosocomial outbreak alerts with the segregated sequences obtained from three co-infected cases: cases 7 (**a**), 6 (**b**) and 10 (**c**). The sequences of the majority- and minority-segregated strains and the mixed sequence of each of the three co-infected cases are indicated

In the case of the eight co-infected patients related to the healthcare setting, it was not initially possible to resolve their epidemiological relationships from genomic analysis, due to the high number of HZ calls in their SARS-CoV-2 sequences, which made it difficult to include them in the corresponding analysis. In six of them, we were able to segregate the co-infecting strains. This allowed us to confirm that three of them were involved in their respective outbreaks (with 7, 13 and 16 cases, respectively, two in January 2021 and one in December 2021, once it had been established that they were infected with the outbreak strain (0–1 SNP with respect to other cases involved in the outbreaks) and another different strain (Fig. 5). Moreover, after segregation of the strains, we were able to rule out that the other three patients were involved in nosocomial outbreaks (more than 5 SNPs with respect to any other case diagnosed within a 2-month time frame).

## Discussion

The main reason why there is so little information on the issue of SARS-CoV-2 co-infection could be the methodological challenge associated with the identification of co-infections when applying genomic analysis, which includes strict quality requirements applied to rule out the possible impact of laboratory cross-contamination, technical problems during sequencing, bioinformatic artefacts or sequencing noise on incorrect allele assignment in calls in the final consensus sequence. This means that whenever a high number of heterozygous (HZ) calls are identified, these sequences are excluded from the analysis. At the same time, the same stringent measures may be responsible for missing SARS-CoV-2 co-infections associated with the presence of HZ calls.

Apart from a few isolated descriptions of SARS-CoV-2 co-infections [20–25], there has been a recent surge of interest in identifying them, coinciding with the first descriptions of SARS-CoV-2 recombinants [8, 9] between the Delta and Omicron lineages. Recombination is common in SARS [26] and obviously requires the simultaneous presence of two different strains in the same individual. However, due to the fact that the first SARS-CoV-2 recombinant alerts involved the Delta and Omicron BA.1 and BA.2 lineages, very few studies have described co-infections prior to this point in time [14, 15]. Most studies have focused on more systematic analyses of mixed infections [11, 13], and many others reporting individual anecdotal cases [20–24] have confined themselves to tracking co-infections involving those lineages and the particular time period when they were co-circulating (mainly end of 2021-first quarter of 2020). As a result of this, analytical approaches designed to identify co-infections have tended to rely on selecting the set of phylogenetically informative SNPs that differentiate between lineages and using them to drive the identification of HZ calls as a proxy for the identification of co-infection. This means that we still do not have an unbiased and more comprehensive view of SARS-CoV-2 co-infection during the pandemic, regardless of the lineages involved. Our study constitutes, to the best of our knowledge, the first systematic analysis of co-infections (i) over the pandemic as a whole, which is (ii) not limited to the most recent highly divergent lineages, and (iii) segregates the strains involved to facilitate their inclusion in any epidemiological or virological characterization.

Rather than selecting possible candidates for co-infection from a curated list of informative SNPs that can only identify co-infections between a limited number of divergent lineages, our analysis is based on the detection of HZ calls, supported by a set of analytical requirements that increase the likelihood of them being robust indicators of co-infection and minimize the possibility of them being the result of sequencing noise or technical artefacts.

As a result of our unbiased approach, we discovered that co-infections have occurred throughout the pandemic, well before and also during the co-circulation of divergent lineages, and even in periods when a majority variant was circulating. These data indicate that recombination may have been a more common driver of SARS-Cov-2 diversification than was supposed. A significant percentage of the co-infections involved strains from the same lineage, which would have been missed in any strategy based on a curated list of selected markers. Our co-infection prevalence was 0.18%, which is similar to the figures (0.2%) obtained for the restricted analysis of divergent lineages in the short time period when they were co-circulating [12]. It should also be noted that our criteria for considering co-infections are much more rigorous than those generally applied, and we may therefore be overlooking some co-infections with strains that are more closely related to each other than the 8-SNP threshold we determined for our study. Moreover, our analysis pipeline is unable to detect co-infections involving minor lineages that constitute less than 25% of the mean total viral population. Overall, the expected frequency of co-infections during the COVID-19 pandemic as a whole seems not be lower than those reported for pandemic time points when co-infections were considered to be most likely.

Our selection of co-infected cases was based not only on the statistical conditions associated with the distribution of allelic frequencies in the HZ calls, designed to ensure that candidates for co-infection corresponded to the presence of two strains, but also on analytical re-confirmations to rule out cross-contamination at any stage from the initial sample handling to the different phases of the sequencing process. Host genetic characterization has only been exceptionally applied in viral genomic analysis in the assignment of SARS-CoV-2 re-infections [4, 5], leading to a probably unacknowledged lack of robustness in the proportion of reported cases that were true re-infections. Something similar occurred in studies focusing on SARS-CoV-2 co-infections: only a minority checked that samples eventually classified as co-infections harboured human material from a single individual [14, 21]. In most studies, precautionary measures are aimed only at ruling out mistakes in the sequencing process by re-sequencing the samples [11, 13]. In our final selection of co-infection cases, we (i) ensured single-host content and (ii) strongly minimize cross-contamination at any stage.

Only a few studies include an analysis of additional longitudinal samples from co-infected cases. These specimens are very useful, not only to confirm the co-infection but also to explore the dynamics of the two strains. In our study, the analysis of additional specimens revealed different behaviours: co-infection persistence, dynamic changes in the frequencies of co-infecting strains or rapid clearance of one of them.

Finally, our validation process was enhanced by including an integrated analysis of our segregated strains. The use of a primer amplicon-based sequencing strategy may introduce amplification bias towards specific lineages (something that would not happen if using metagenomic approaches). This could result in inaccurate determination of the proportions and segregation of strains detected in co-infections. Consequently, downstream analyses that rely on strain segregation could be affected if such amplification bias occurs. However, based on the in vitro validation approach we performed, this potential bias is not expected to significantly affect the mean proportion of both strains involved in the co-infection.

Once segregated the sequences involved in co-infections, we then followed a series of validation stages. First, we integrated the segregated sequences with sequences obtained from strains circulating in the same time windows as our candidate cases. By doing this, we were able to identify inconsistent behaviour in some of the segregated co-infecting strains, which allowed us to reinterpret a number of cases with HZ calls that might otherwise have been mistaken for co-infections as sequencing noise (most due to high *C*_*t*_ values). Phylogenetic analysis of the segregated strains also highlighted that some candidate strains that had passed all the preceding analytical filters were actually microevolutions that had resulted in (i) within-host maintenance of a lineage that had already been ousted from the population and (ii) sufficiently high within-host diversity acquisition to be misinterpreted as a co-infection. It has been reported that the evolutionary rate for SARS-CoV-2 in severely immunosuppressed patients may be higher than the average rate observed in the population setting [6, 7].

There is no clear pattern in patients with co-infection. We found no apparent predisposing reasons for co-infection, since half of the patients had no medical history of interest. Regarding the clinical presentation of the COVID-19 episode, there were patients with both mild/asymptomatic and severe symptoms. The question remains whether vaccination has a protective effect on co-infection, as half of our co-infections occurred in the pre-vaccination era. Interestingly, almost 60% of the patients were healthcare-related, either in the form of a nosocomial outbreak or as healthcare workers. The number of cases with co-infection makes statistical comparisons difficult, but the data suggest a probable link between this patient profile and co-infection, presumably due to higher exposure to different infectious cases of SARS-CoV-2 in the hospital setting.

It is not easy to fully explain the reasons for the co-infections and assess whether they are due either to a superinfection from a new exposure before the first infection has been resolved or to a primary co-infection due to exposure to another co-infected case. Superinfections may have occurred when the incidence rates were highest during the pandemic, mainly in cases at high risk of overexposure (nosocomial outbreaks, HCWs, highly dependent cases) as was the case in our study [27]. Increased risk for co-infection involving different lineages (Delta, Omicron BA.1 or Omicron BA.2) has been reported for in immunocompromised patients elsewhere [28]. In five of our cases, one of the co-infecting strains was also identified circulating in the population close to the case diagnosis (data not shown), which would probably indicate superinfection. In other studies, superinfections have also been found by detecting other household members infected with just one of the two strains or associated with haemodialysis cases, more frequently exposed to the nosocomial setting [25].

An analysis of SARS-CoV-2 co-infections should not only be confined to identification, but also offer the possibility of including co-existing strains in the virological or epidemiological analysis performed systematically on all sequenced cases. This means separating the two sequences to minimize the proportion of cases that cannot be included in the analysis, something that has not been done in other studies focused on SARS-CoV-2 co-infections. Our study developed a pipeline to segregate the sequences from all candidates for co-infection with relative proportions not close to 50:50, which are non-segregable. As a result, we were able to add 22 new sequences to our population-based analysis, assign lineages and include them in our systematic epidemiological analysis. Indeed, thanks to segregation, we ruled in or ruled out new cases linked to nosocomial outbreaks in which analysis was impaired by the presence of HZ calls. The inclusion of each and every case involved in SARS-CoV-2 outbreaks proved essential to reveal the true complexity of their transmission, which would not otherwise have been suspected without genomic support [29]. Beyond the analysis of outbreaks, other relevant questions, such as distinguishing between re-infection and persistence, the assignment of new lineages imported into a population or the identification of lineages that are refractory to some anti-COVID-19 treatments, would all be impaired in co-infected patients if the co-infecting strains were not segregated. The strategy on which our segregation pipeline is based could be adapted to other pathogens where co-infection identification and sequence segregation is relevant. We are currently adapting it to tuberculosis for application in prisons and high incidence settings where co-infections are expected.

## Conclusions

In summary, we have developed a bioinformatic algorithm that makes it possible to identify SARS-CoV-2 co-infection events, regardless of whether or not the strains involved belong to the same or different lineages. Strict application of a series of validations eliminated candidates due to sequencing noise and laboratory cross-contamination, finally resulting in a set of robustly confirmed co-infections. Our pipeline further allowed segregation of the two co-infecting strains when they were distributed within the patient in a ratio of at least 75:25, allowing the exploitation of genomic data from cases whose epidemiological analysis was impaired prior to segregation. Our data demonstrated that it was possible to identify co-infection cases throughout the pandemic, not just in the time periods when highly divergent lineages were co-circulating, and to determine that co-infections involving different lineages or strains from the same lineage were occurring in the same proportion. Most cases were mild, did not require medical assistance and were not vaccinated. Notably, a large proportion of patients were associated with the hospital environment, either because they were involved in nosocomial outbreaks or because they were HCWs and therefore more likely to be overexposed to infectious cases. To complete the limited systematic information available on SARS-CoV-2 co-infection, further studies are needed that cover the entire COVID-19 pandemic, are not restricted to selected variants and are supported by rigorous case confirmation procedures, ideally segregating and analysing the strains involved.

## Supplementary Information

**Additional file 1: Fig. S1.** Multiple alignment of sequences obtained from the segregated majority strain from patient 40. Positions corresponding to the consensus core genome shared by all sequences are in dark blue. light blue. red and green. Positions corresponding to the specific diversity observed in each specimen are in grey. **Table S1.** Results obtained from applying the co-infection pipeline to Alpha/Delta mix. **Table S2.** Results obtained from applying the co-infection pipeline to Omicron BA.2/Omicron BA.5 mix. **Table S3.** Results obtained from applying the co-infection pipeline to Omicron BA.5/Omicron BA.5 mix. **Table S4.** Sequence/specimen characteristics of candidate SARS-CoV-2 co-infections. The first 14 patients correspond to co-infections that were finally validated; the remaining 46 cases are candidates that were discarded during the various validation steps. *Lineage assigned after discarding segregated positions with lower confidence.

## Data Availability

The data (fastq files) supporting the findings of this study are available at ENA under the project name PRJEB58615 (https://www.ebi.ac.uk/ena/browser/view/PRJEB58615).
