# Supplementary material for: Systematic genomic analysis of SARS-CoV-2 co-infections throughout the pandemic and segregation of the strains involved
Source: Genome Med. 2023 Jul 24;15:57. doi: 10.1186/s13073-023-01198-z (PMC10367318; doi:10.1186/s13073-023-01198-z)
Supplement: Supplementary file 1 — Additional file 1: Fig. S1. Multiple alignment of sequences obtained from the segregated majority strain from patient 40. Positions corresponding to the consensus core genome shared by all sequences are in dark blue. light blue. red and green. Positions corresponding to the specific diversity observed in each specimen are in grey. Table S1. Results obtained from applying the co-infection pipeline to Alpha/Delta mix. Table S2. Results obtained from applying the co-infection pipeline to Omicron BA.2/Omicron BA.5 mix. Table S3. Results obtained from applying the co-infection pipeline to Omicron BA.5/Omicron BA.5 mix. Table S4. Sequence/specimen characteristics of candidate SARS-CoV-2 co-infections. The first 14 patients correspond to co-infections that were finally validated; the remaining 46 cases are candidates that were discarded during the various validation steps. *Lineage assigned after discarding segregated positions with lower confidence. [file 13073_2023_1198_MOESM1_ESM.docx]

**
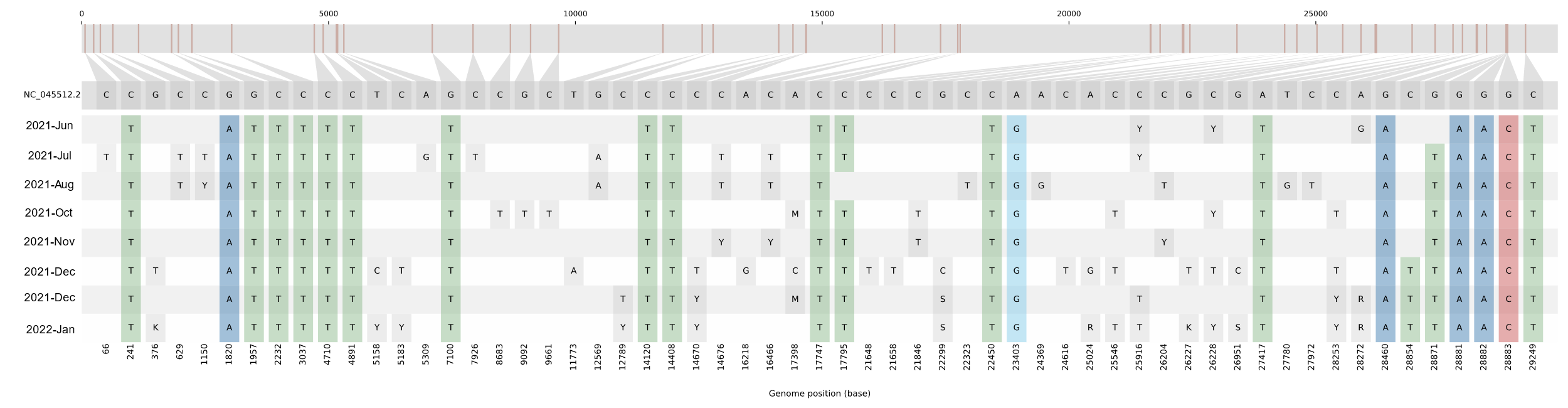
**

**Fig S1. Multiple alignment of sequences obtained from the segregated majority strain from patient 40 (8 specimens obtained during the persistence period; dates on the left).** Positions corresponding to the consensus core genome shared by all sequences are in dark blue. light blue. red and green. Positions corresponding to the specific diversity observed in each specimen are in grey.

**Table S1**. Results obtained from applying the co-infection pipeline to Alpha/Delta mix.

|  |  | **Majority: Alpha (B.1.1.7) /Minority: Delta (AY.43)** | | | | | |
| --- | --- | --- | --- | --- | --- | --- | --- |
|  |  | **Positive controls** | | | **Negative controls** | | |
| **Expected** | Mix ratio | 1:1 | 2:1 | 3:1 | 9:1 | 1:0 | 0:1 |
|  | Expected mean major alleles proportion (%) | 50% | 66% | 75% | Below detection limit | No mix | No mix |
|  | Expected number HZ calls | 64 | 64 | 64 |  | No mix | No mix |
| **Experimental** | Mean major alleles proportion (%) | 54% | 66% | 77% | 82% | 0% | 0% |
|  | Number HZ calls | 65 | 65 | 63 | 4 | 0 | 0 |
|  | SD of major alleles (%) | 3% | 5% | 4% | 0% | 0% | 0% |
|  | % SNPs within SD | 86% | 88% | 90% | 100% | 0% | 0% |
| **Segregation** | Majority lineage (conflict %) | B.1.617.2 (0%) | **B.1.1.7 (0%)** | **B.1.1.7 (0%)** |  |  |  |
|  | Minority lineage (conflict %) | B.1.1 (0%) | **AY.43 (0%)** | **AY.43 (0%)** |  |  |  |

**Table S2**. Results obtained from applying the co-infection pipeline to Omicron BA.2/Omicron BA.5 mix.

|  |  | **Majority: Omicron (BA.2) /Minority: Omicron (BA.5.1)** | | | | | |
| --- | --- | --- | --- | --- | --- | --- | --- |
|  |  | **Positive controls** | | | **Negative controls** | | |
| **Expected** | Mix ratio | 1:1 | 2:1 | 3:1 | 9:1 | 1:0 | 0:1 |
|  | Expected mean major alleles proportion (%) | 50% | 66% | 75% | Below detection limit | No mix | No mix |
|  | Expected number HZ calls | 16 | 16 | 16 |  | No mix | No mix |
| **Experimental** | Mean major alleles proportion (%) | 54% | 62% | 74% | 0% | 0% | 0% |
|  | Number HZ calls | 16 | 16 | 16 | 0 | 0 | 0 |
|  | SD of major alleles (%) | 3% | 4% | 4% | 0% | 0% | 0% |
|  | % SNPs within SD | 81% | 75% | 88% | 0% | 0% | 0% |
| **Segregation** | Majority lineage (conflict %) | BA.5.1 (40%) | **BA.2 (0%)** | **BA.2 (0%)** |  |  |  |
|  | Minority lineage (conflict %) | BA.2 (0%) | **BA.5.1 (0%)** | **BA.5.1 (0%)** |  |  |  |

**Table S3**. Results obtained from applying the co-infection pipeline to Omicron BA.5/Omicron BA.5 mix.

|  |  | **Majority: Omicron (BA.5.1) /Minority: Omicron (BA.5.1.23)** | | | | | |
| --- | --- | --- | --- | --- | --- | --- | --- |
|  |  | **Positive controls** | | | **Negative controls** | | |
| **Expected** | Mix ratio | 1:1 | 2:1 | 3:1 | 9:1 | 1:0 | 0:1 |
|  | Expected mean major alleles proportion (%) | 50% | 66% | 75% | Below detection limit | No mix | No mix |
|  | Expected number HZ calls | 9 | 9 | 9 |  | No mix | No mix |
| **Experimental** | Mean major alleles proportion (%) | 53% | 63% | 74% | 0% | 0% | 0% |
|  | Number HZ calls | 9 | 9 | 9 | 0 | 0 | 0 |
|  | SD of major alleles (%) | 2% | 2% | 2% | 0% | 0% | 0% |
|  | % SNPs within SD | 100% | 100% | 89% | 0% | 0% | 0% |
| **Segregation** | Majority lineage (conflict %) | BA.5.1.23 (0%) | **BA.5.1 (0%)** | **BA.5.1 (0%)** |  |  |  |
|  | Minority lineage (conflict %) | BA.5.1 (33%) | **BA.5.1.23 (0%)** | **BA.5.1.23 (0%)** |  |  |  |

**Table S4. Sequence/specimen characteristics of candidate SARS-CoV-2 co-infections.** The first 14 patients correspond to co-infections that were finally validated; the remaining 46 cases are candidates that were discarded during the various validation steps. *Lineage assigned after discarding segregated positions with lower confidence.

| **Patient** | **GenN2 Ct** | **COV>30X** | **Total HZ** | **MHP** | **SHP** | **SWS** | **Minority pangolin** | **Majority pangolin** | **Date** | **Reason for exclusion** |
| --- | --- | --- | --- | --- | --- | --- | --- | --- | --- | --- |
| 1 | n.a | 99.69 | 12 | 0.66 | 0.04 | 0.83 | A.5 | B.1 | 2020-03-09 |  |
| 2 | n.a | 99.08 | 11 | 0.64 | 0.03 | 0.91 | A.5 | B.1 | 2020-03-09 |  |
| 3 | n.a | 99.02 | 8 | 0.73 | 0.03 | 0.88 | B.1 | B.1 | 2020-04-08 |  |
| 4 | 20 | 99.66 | 8 | 0.66 | 0.04 | 0.75 | B.1.177 | B.1.177 | 2020-08-11 |  |
| 5 | 25 | 98.71 | 9 | 0.54 | 0.03 | 1 | B.1.177 | B.1.177 | 2020-11-01 |  |
| 6 | 31 | 99.02 | 37 | 0.6 | 0.05 | 0.76 | B.1.177 | B.1 | 2021-01-18 |  |
| 7 | 18 | 99.67 | 19 | 0.61 | 0.04 | 0.84 | B.1.177 | B.1.177 | 2021-01-21 |  |
| 8 | 25 | 98.79 | 30 | 0.55 | 0.06 | 0.9 | B.1.617.2 | B.1.617.2 | 2021-08-08 |  |
| 9 | 26 | 99.67 | 15 | 0.58 | 0.03 | 0.93 | AY.5.5 | AY.43 | 2021-08-25 |  |
| 10 | 12 | 98.44 | 9 | 0.71 | 0.03 | 0.89 | BA.1.17 | BA.1.1.1 | 2021-12-14 |  |
| 11 | 16 | 99.62 | 85 | 0.71 | 0.07 | 0.74 | AY.121 | BA.1 | 2021-12-17 |  |
| 12 | 31 | 98.91 | 11 | 0.73 | 0.04 | 0.73 | BA.1.1 | BA.1.1 | 2022-02-11 |  |
| 13 | 14 | 99.3 | 12 | 0.57 | 0.07 | 0.92 | BA.2 | BA.2 | 2022-05-12 |  |
| 14 | 18 | 98.61 | 22 | 0.7 | 0.05 | 0.82 | BA.5* | BE.1.1 | 2022-06-27 |  |
| 15 | n.a | 96.53 | 30 | 0.73 | 0.06 | 0.73 | B.1.1 | B.1.1 | 2020-04-02 | phylogenetic inconsistency |
| 16 | n.a | 99.13 | 13 | 0.74 | 0.08 | 0.77 | B | B | 2020-04-16 | phylogenetic inconsistency |
| 17 | n.a | 94.49 | 17 | 0.74 | 0.08 | 0.76 | B.1 | B.1 | 2020-05-27 | phylogenetic inconsistency |
| 18 | n.a | 95.79 | 22 | 0.73 | 0.06 | 0.77 | B.1 | B.1 | 2020-06-09 | phylogenetic inconsistency |
| 19 | 18 | 99.08 | 9 | 0.73 | 0.08 | 0.78 | A.5 | A.5 | 2020-06-22 | phylogenetic inconsistency |
| 20 | 23 | 99.09 | 10 | 0.74 | 0.07 | 0.7 | B.1 | B.1 | 2020-07-13 | phylogenetic inconsistency |
| 21 | 30 | 90.66 | 13 | 0.73 | 0.07 | 0.77 | B.1.1.7 | B.1.1.7 | 2021-03-12 | phylogenetic inconsistency |
| 22 | 30 | 92.23 | 52 | 0.74 | 0.08 | 0.75 | B.1.1.7 | B.1.1.7 | 2021-03-30 | phylogenetic inconsistency |
| 23 | 30 | 95.94 | 18 | 0.72 | 0.07 | 0.72 | B.1.1.7 | B.1.1.7 | 2021-05-12 | phylogenetic inconsistency |
| 24 | n.a | 97.44 | 50 | 0.74 | 0.08 | 0.78 | B.1.621 | B.1.633 | 2021-06-11 | phylogenetic inconsistency |
| 25 | 19 | 99.58 | 15 | 0.74 | 0.08 | 0.73 | P.1 | P.1 | 2021-06-16 | phylogenetic inconsistency |
| 26 | 28 | 98.08 | 34 | 0.69 | 0.07 | 0.85 | AY.5.5 | AY.70 | 2021-06-24 | not confirmed |
| 27 | 30 | 99.66 | 60 | 0.61 | 0.07 | 0.72 | B.1.621 | XB | 2021-06-30 | not confirmed |
| 28 | 20 | 99.65 | 69 | 0.57 | 0.04 | 0.87 | AY.124 | B.1.1.7 | 2021-07-06 | not confirmed |
| 29 | 27 | 97.13 | 21 | 0.74 | 0.08 | 0.76 | B.1.1 | P.1 | 2021-07-13 | phylogenetic inconsistency |
| 30 | 18 | 99.64 | 36 | 0.54 | 0.03 | 0.89 | AY.73 | B.1.617.2 | 2021-07-16 | not confirmed |
| 31 | 18 | 95.73 | 19 | 0.67 | 0.04 | 0.89 | AY.44 | AY.5.5 | 2021-07-20 | not confirmed |
| 32 | 21 | 99.66 | 19 | 0.73 | 0.06 | 0.89 | AY.122 | AY.122 | 2021-07-22 | phylogenetic inconsistency |
| 33 | 23 | 95.89 | 28 | 0.7 | 0.07 | 0.75 | AY.42 | AY.53 | 2021-07-24 | not confirmed |
| 34 | 18 | 96.52 | 11 | 0.56 | 0.03 | 1 | AY.43 | AY.42 | 2021-07-26 | not confirmed |
| 35 | 25 | 93.38 | 61 | 0.6 | 0.08 | 0.85 | B.1 | B.1.617.2 | 2021-07-26 | not confirmed |
| 36 | 27 | 98.57 | 60 | 0.67 | 0.08 | 0.7 | B.1.1.7 | AY.43 | 2021-07-26 | not confirmed |
| 37 | 29 | 91.54 | 44 | 0.64 | 0.08 | 0.8 | AY.43 | B.1.617.2 | 2021-08-16 | not confirmed |
| 38 | 33 | 94.76 | 35 | 0.73 | 0.08 | 0.77 | B.1.177 | AY.43 | 2021-08-20 | not confirmed |
| 39 | n.a | 91.14 | 15 | 0.73 | 0.08 | 0.8 | B.1.617.2 | AY.5 | 2021-08-25 | not confirmed |
| 40 | 23 | 99.58 | 39 | 0.58 | 0.05 | 0.82 | B.1.1 | B.1.1 | 2021-12-22 | epidemiological inconsistency |
| 41 | 24 | 99.66 | 22 | 0.58 | 0.08 | 0.86 | B.1.177 | B.1.177 | 2021-10-08 | epidemiological inconsistency |
| 42 | 29 | 95.82 | 10 | 0.7 | 0.07 | 0.9 | AY.122 | AY.122 | 2021-10-23 | phylogenetic inconsistency |
| 43 | 28 | 91.12 | 14 | 0.69 | 0.08 | 0.71 | B.1.617.2 | B.1.617.2 | 2021-11-17 | phylogenetic inconsistency |
| 44 | 22 | 98.19 | 9 | 0.57 | 0.08 | 0.89 | BA.1.17 | BA.1.18 | 2021-12-20 | not confirmed |
| 45 | 14 | 98.33 | 8 | 0.67 | 0.07 | 0.75 | BA.1.1.1 | BA.1.17 | 2022-01-05 | not confirmed |
| 46 | 27 | 99.59 | 8 | 0.71 | 0.04 | 0.75 | BA.1.1.1 | BA.1.17 | 2022-02-08 | not confirmed |
| 47 | 15 | 99.45 | 24 | 0.71 | 0.08 | 0.71 | BA.2 | BA.1.1 | 2022-02-13 | not confirmed |
| 48 | 25 | 99.62 | 45 | 0.54 | 0.04 | 0.96 | BA.2 | BA.2 | 2022-02-17 | not confirmed |
| 49 | 26 | 99.54 | 13 | 0.64 | 0.05 | 0.85 | BA.1.17 | BA.1.17 | 2022-02-18 | not confirmed |
| 50 | 24 | 99.52 | 13 | 0.71 | 0.07 | 0.85 | BA.1.17 | BA.1.17 | 2022-02-19 | not confirmed |
| 51 | 36 | 99.53 | 10 | 0.74 | 0.04 | 0.7 | BA.1.1 | BA.1.17 | 2022-02-20 | not available |
| 52 | 23 | 98.95 | 8 | 0.74 | 0.07 | 0.75 | BA.2 | BA.2 | 2022-03-28 | phylogenetic inconsistency |
| 53 | 30 | 97.63 | 30 | 0.74 | 0.07 | 0.8 | BA.2 | BA.2 | 2022-04-06 | phylogenetic inconsistency |
| 54 | 27 | 97.1 | 9 | 0.71 | 0.05 | 0.89 | BA.2.9 | BA.2.9 | 2022-04-08 | phylogenetic inconsistency |
| 55 | 30 | 97.3 | 12 | 0.71 | 0.08 | 0.83 | BA.2 | BA.2 | 2022-05-09 | phylogenetic inconsistency |
| 56 | 25 | 97.44 | 9 | 0.67 | 0.07 | 0.89 | BA.2.3 | BA.2 | 2022-05-10 | not confirmed |
| 57 | 20 | 99.02 | 28 | 0.73 | 0.08 | 0.86 | BA.2 | BA.2 | 2022-05-29 | phylogenetic inconsistency |
| 58 | 22 | 98.88 | 27 | 0.69 | 0.08 | 0.74 | BA.2 | BA.5.1 | 2022-06-06 | phylogenetic inconsistency |
| 59 | 21 | 98.56 | 20 | 0.67 | 0.06 | 0.85 | BE.1.1 | BA.4.1 | 2022-07-10 | not confirmed |
| 60 | 25 | 99.52 | 27 | 0.6 | 0.07 | 0.81 | BA.5.1 | BA.2 | 2022-07-24 | not confirmed |
| 61 | 26 | 98.02 | 21 | 0.73 | 0.08 | 0.76 | BA.2 | BA.5.2.1 | 2022-08-21 | not confirmed |
| 62 | 28 | 97.12 | 8 | 0.65 | 0.05 | 0.88 | BA.2 | BA.2 | 2022-09-08 | epidemiological inconsistency |
